# SARS Coronavirus Detection Methods

**DOI:** 10.3201/eid1107.041045

**Published:** 2005-07

**Authors:** Susanna K.P. Lau, Xiao-Yan Che, Patrick C.Y. Woo, Beatrice H.L. Wong, Vincent C.C. Cheng, Gibson K.S. Woo, Ivan F.N. Hung, Rosana W.S. Poon, Kwok-Hung Chan, J.S. Malik Peiris, Kwok-Yung Yuen

**Affiliations:** * University of Hong Kong, Hong Kong Special Administrative Region, People's Republic of China;; † First Military Medical University, Guangzhou, People's Republic of China;; ‡ Queen Mary Hospital, Hong Kong Special Administrative Region, People's Republic of China

**Keywords:** SARS-Cov, qRT-PCR, nucleocapsid, capture ELISA

## Abstract

Using clinical samples from patients with severe acute respiratory syndrome, we showed that the sensitivities of a quantitative reverse transcription–polymerase chain reaction (80% for fecal samples and 25% for urine samples) were higher than those of the polyclonal (50% and 5%) and monoclonal (35% and 8%) antibody-based nucleocapsid antigen capture enzyme-linked immunosorbent assays.

The epidemic of severe acute respiratory syndrome (SARS) in 2003, caused by SARS-associated coronavirus (SARS-CoV), has affected 30 countries, with 8,098 cases and 774 deaths (1–8). Early diagnosis of SARS-CoV infection, which involves viral detection, is important for preventing future epidemics. Since culturing of SARS-CoV is difficult and insensitive, the reverse transcription–polymerase chain reaction (RT-PCR) and quantitative RT-PCR (qRT-PCR) has been the working standard in diagnosis (2,9). Nevertheless, these techniques are relatively expensive and rely on the availability of equipment and expertise. We recently reported the development of 2 sandwich enzyme-linked immunosorbent assays (ELISAs) for detection of SARS-CoV nucleocapsid protein in clinical specimens of SARS patients (10,11). However, no studies have been conducted to compare the sensitivities of ELISA with those of RT-PCR. Although PCR assays are generally more sensitive, ELISAs are less expensive and easier to conduct (12,13). To evaluate the potential usefulness of ELISA in diagnosing SARS-CoV infections, we compared the performance of ELISA and qRT-PCR and studied the correlation between their results.

## The Study

Fecal specimens (n = 40, from 40 patients 1–27 days after symptom onset) and urine specimens (n = 133, from 101 patients 2–57 days after symptom onset) were collected from SARS patients hospitalized in Hong Kong from March to May 2003. SARS was confirmed by the presence of serum immunoglobulin (Ig) G against SARS-CoV by an immunofluorescence assay (4). Specimens were tested with polyclonal and monoclonal antibody–based capture ELISAs for SARS-CoV nucleocapsid protein and real-time qRT-PCR. Control urine (n = 100) and fecal (n = 100) specimens were obtained from hospitalized patients without SARS.

SARS-CoV nucleocapsid protein was detected by polyclonal antibody–based ELISA according to published protocols (7,11). SARS-CoV nucleocapsid protein was detected by monoclonal antibody–based ELISA using a modified protocol for serum samples (10). Briefly, fecal and urine specimens were inactivated with 2% and 0.5% phenol, respectively, for 15 min before centrifugation and dilution in phosphate-buffered saline with 2% skim milk. One hundred microliters of 1:10 diluted fecal specimens or 1:2 diluted urine specimens was added to wells previously coated with antinucleocapsid monoclonal antibodies. Plates were incubated, washed, treated with antinucleocapsid rabbit monoclonal antibodies, and analyzed as described previously (10,11). RNA extraction and real-time qPCR assay specific for the 1b region of SARS-CoV were conducted as described previously (3,9).

We compared the detection rates of 2 ELISAs and real-time qRT-PCR using the McNemar test and studied the correlation between the optical density values at 450 nm (OD_450_) of the 2 ELISAs and log_10_ viral concentrations, as determined by real-time qRT-PCR, by linear regression (SPSS version 11.0, SPSS Inc., Chicago, IL, USA). A p value <0.05 was regarded as significant.

A comparison of the 2 ELISAs is shown in the Figure and Table 1. The cutoffs of the polyclonal antibody–based ELISA have been determined previously, with specificities of 96% and 99% for fecal and urine specimens, respectively (11). The baselines of the monoclonal antibody–based ELISA were determined by using 100 control fecal and urine specimens, with mean OD_450_ values of 0.089 and 0.05 and standard deviation (SD) values of 0.074 and 0.03, respectively. The specificities of the monoclonal antibody–based ELISA were 93% for fecal specimens and 98% for urine specimens, as determined using cutoffs defined as the mean + 2 SD. Of 40 fecal samples obtained from SARS patients, 20 (50%) obtained on days 9 to 23 after onset of symptoms were positive by the polyclonal antibody–based ELISA, and 14 (35%) obtained on days 2 to 21 were positive by the monoclonal antibody–based ELISA. Of 133 urine samples, 6 (5%) obtained on days 16 to 32 after onset of symptoms were positive by the polyclonal antibody–based ELISA, and 11 (8%) obtained on days 6 to 45 were positive by the monoclonal antibody–based ELISA. Results of the polyclonal antibody–based ELISA were comparable with our previous data on different specimens (11). The OD_450_ values of both fecal (Pearson correlation 0.610, p<0.0005) and urine specimens (Pearson correlation 0.475, p<0.0005) detected by the 2 ELISAs were significantly correlated.

**Figure:**
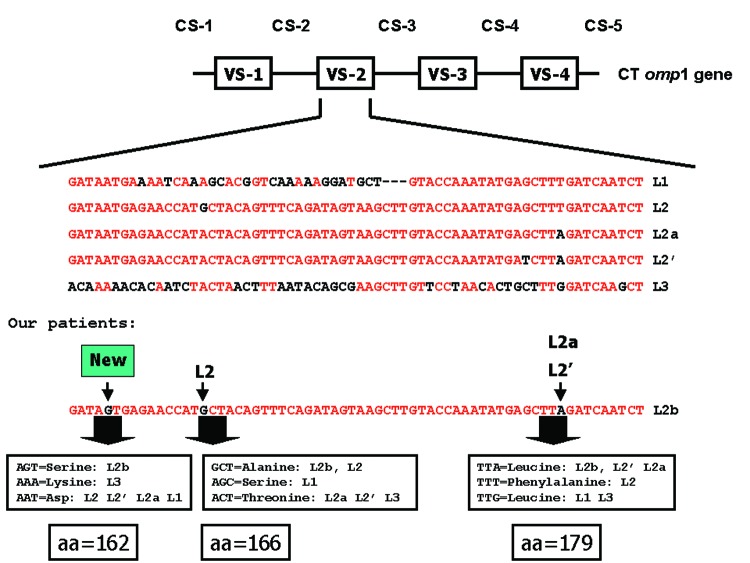
Evaluation of polyclonal and monoclonal antibody–based enzyme-linked immunosorbent assays (ELISAs) for detecting nucleocapsid protein in fecal and urine specimens. The dashed horizontal lines represent the corresponding cutoff optical density values at 450 nm (OD_450_). SARS, severe acute respiratory syndrome.

**Table 1:** Detection of SARS-CoV in clinical specimens by qRT-PCR and ELISA in relation to time from onset of symptoms*

Days from onset of symptoms	No. specimens	No. positive specimens (%)		
		qRT-PCR	Polyclonal antibody–based ELISA	Monoclonal antibody–based ELISA
Fecal specimens				
1–5	4	3 (75)	0	1 (25)
6–10	4	3 (75)	2 (50)	0
11–15	13	9 (69)	7 (54)	5 (38)
16–20	14	13 (93)	9 (64)	7 (50)
21–25	4	3 (75)	2 (50)	1 (25)
26–30	1	1 (100)	0	0
Urine specimens				
1–5	1	0	0	0
6–10	13	1 (8)	0	2 (15)
11–15	19	3 (16)	0	0
16–20	67	24 (36)	5 (7)	7 (10)
21–25	10	1 (10)	0	1 (10)
26–30	11	2 (18)	0	0
31–40	5	0	1 (20)	0
41–50	5	2 (40)	0	1 (20)
51–60	2	0	0	0

*SARS-CoV, severe acute respiratory syndrome coronavirus; qRT-PCR, quantitative reverse transcription–polymerase chain reaction; ELISA, enzyme-linked immunosorbent assay.

## Conclusions

The method of choice for early diagnosis of SARS-CoV infection should be the qRT-PCR. The sensitivity of qRT-PCR is superior to that of both ELISAs. Moreover, qRT-PCR can detect SARS-CoV earlier in fecal specimens (Tables 1 and 2). Among the 40 fecal samples from SARS patients, 32 (80%) were positive by qRT-PCR, which was significantly higher than that of the polyclonal (50%) and monoclonal (35%) antibody-based ELISAs (McNemar test, p<0.005 and p<0.001, respectively). Of the 133 urine samples from SARS patients, 33 (25%) were positive by qRT-PCR, which was also significantly higher than that of the polyclonal (5%) and monoclonal (8%) antibody-based ELISAs (McNemar test, p<0.001 for both comparisons). When qRT-PCR was used as a standard, the sensitivities of the polyclonal and monoclonal antibody–based ELISAs were 53.1% (17/32) and 43.8% (14/32) in fecal specimens, and 12.1% (4/33) and 15.2% (5/33) in urine specimens, respectively. The qRT-PCR can detect SARS-CoV in fecal specimens obtained on days 1 to 27 after onset of symptoms and in urine specimens obtained on days 9 to 45. Moreover, 6 (75%) of the 8 fecal specimens obtained on days 1 to 10 were positive by qRT-PCR. All 3 tests had the highest detection rates in fecal specimens collected on days 16 to 20, which suggested that this was the period of peak viral shedding in stool. The detection rates in urine specimens were much lower than those in fecal specimens in all 3 assays.

**Table 2:** Detection of SARS-CoV by qRT-PCR and ELISA in clinical specimens of patients with SARS*

RNA concentration (copies/mL)	Fecal specimens			Urine specimens		
	No. specimens	No. positive by polyclonal antibody–based ELISA	No. positive by monoclonal antibody–based ELISA	No. specimens	No. positive by polyclonal antibody–based ELISA	No. positive by monoclonal antibody–based ELISA
<3 × 10^2^	8	3	0	100	2	6
3 × 10^2^–<10^4^	5	3	3	16	1	1
10^4^–<10^6^	3	0	0	10	1	1
10^6^–<10^8^	9	5	0	7	2	3
10^8^–<10^10^	13	8	10	0	0	0
≥10^10^	2	1	1	0	0	0
Total	40	20	14	133	6	11

*SARS-CoV, severe acute respiratory syndrome coronavirus; qRT-PCR, quantitative reverse transcription–polymerase chain reaction; ELISA, enzyme-linked immunosorbent assay.

SARS-CoV can be detected during the late phase of illness. Since SARS-CoV cannot be readily isolated from SARS patients after week 3 of illness (14), the detection of SARS-CoV beyond this time may be due to prolonged shedding of nonviable viruses in these patients or the presence of neutralizing immunoglobulins in clinical specimens, which has prevented viral replication in cell cultures.

SARS-CoV RNA concentration and ELISA results were correlated. Higher detection rates by both ELISAs were found in specimens with higher viral concentrations (Table 2). There was also a significant correlation between viral load and ELISA OD_450_ values in fecal specimens tested with the monoclonal antibody–based ELISA (Pearson correlation 0.424, p = 0.003), and in urine specimens tested with both the polyclonal and monoclonal antibody–based ELISAs (Pearson correlation 0.386 and 0.331, respectively, p<0.0005 in both analysis). Although the correlation between viral load and ELISA OD_450_ values in fecal specimens tested with the polyclonal antibody–based ELISA was not significant, there was a trend for such a correlation (Pearson correlation 0.229, p = 0.078).

In this study, fecal and urine samples were used because they are easier and safer to obtain and more readily available. In our previous reports, nucleocapsid protein was detected by the polyclonal antibody–based ELISA in 83% of nasopharyngeal aspirates collected on days 11 to 15 after symptom onset and by the monoclonal antibody–based ELISA in 85% of serum obtained during the first 10 days (10,11). These findings suggest that ELISA may be more useful when used with nasopharyngeal aspirate and serum specimens. However, these specimens were not included in the current study because only small amounts were available. Similar studies should be conducted if such samples are available.

This study was supported by the Research Grant Council Grant (HKU 7532/03M); Vice-Chancellor SARS Research Fund (21395035/39839/20700/420/01 and 21395061/27944/20700/420/01), The University of Hong Kong; and Suen Chi Sun Charitable Foundation SARS Research Fund.
